# Supplementary material for: Relation of urinary bisphenol concentration and diabetes or prediabetes in French adults: A cross-sectional study
Source: PLoS One. 2023 Mar 30;18(3):e0283444. doi: 10.1371/journal.pone.0283444 (PMC10062552; doi:10.1371/journal.pone.0283444)
Supplement: S1 Table — Results are presented as mean (± standard error) for continuous variables and as number (percentage) for categorical variables. (DOCX) [file pone.0283444.s001.docx]

Relation of urinary bisphenol concentration and diabetes or prediabetes in French adults: a cross-sectional study

**Table S1.** **Characteristics of the two populations.**

|  | **Esteban population**  **N = 2,345** | **Bisphenol population**  **N = 852** |
| --- | --- | --- |
| **Age** in years (± SD) | 51.4 (± 13.3) | 51.6 (± 0.4) |
| **Sex** |  |  |
| Men | 1,025 (43.7%) | 385 (45.2%) |
| Women | 1,320 (56.3%) | 467 (54.8%) |
| **Education level** |  |  |
| Less than high school | 648 (27.6%) | 232 (27.2%) |
| High school graduate | 453 (19.3%) | 180 (21.1%) |
| Bachelor’s degree | 581 (24.8%) | 217 (25.5%) |
| Master or PhD | 663 (28.3%) | 223 (26.2%) |
| **Body mass index** |  |  |
| Normal: < 25 kg/m² | 1,214 (51.9%) | 444 (52.2%) |
| Overweight: 25-30 kg/m² | 758 (32.4%) | 276 (32.5%) |
| Obesity: > 30 kg/m² | 367 (15.7%) | 130 (15.3%) |
| **High blood pressure** |  |  |
| Yes | 736 (31.4%) | 277 (32.5%) |
| No | 1,380 (58.8%) | 487 (57.2%) |
| Missing | 229 (9.8%) | 88 (10.3%) |
| **Hypercholesterolemia** |  |  |
| Yes | 664 (28.3%) | 240 (28.2%) |
| No | 1,378 (58.8%) | 488 (57.3%) |
| Missing | 303 (12.9%) | 124 (14.6%) |
| **Sedentariness** |  |  |
| Low | 274 (12.3%) | 102 (12.5%) |
| Moderate | 1,069 (47.6%) | 389 (47.5%) |
| High | 905 (40.3%) | 328 (40.0%) |
| **Duration of smoking** |  |  |
| Never smoker | 1,238 (53.5%) | 434 (51.6%) |
| Less than 20 years | 691 (29.9%) | 258 (30.7%) |
| More than 20 years | 386 (16.7%) | 149 (17.7%) |
| **Energy intake** in kcal/day (± SD) | 1,966.9 (± 593.8) | 1,981.3 (± 611.8) |
| **Diabetes** | 157 (6.7%) | 62 (7.3%) |
| **Prediabetes** | 239 (10.2%) | 90 (10.6%) |
| **Diabetes or prediabetes** | 396 (16.9%) | 152 (17.8%) |
| **No diabetes** | 1949 (83,1%) | 700 (82,2%) |

Results are presented as mean (± standard error) for continuous variables and as number (percentage) for categorical variables.
