# Supplementary material for: Relation of urinary bisphenol concentration and diabetes or prediabetes in French adults: A cross-sectional study
Source: PLoS One. 2023 Mar 30;18(3):e0283444. doi: 10.1371/journal.pone.0283444 (PMC10062552; doi:10.1371/journal.pone.0283444)
Supplement: Attachment — Submitted filename: Delpierre PLOSONE D 22 22640.docx [file pone.0283444.s002.docx]

This is a cross-sectional study of the association between the bisphenols BPA, BPS and BPF with (diabetes + prediabetes) in 852 adults living in France. The authors find a detrimental association for BPA but no association for BPS nor for BPF.

Do you get the same results if you study just diabetes, prediabetes compared to `normal glucose tolerance`, using multinomial regression; or for diabetes vs (prediabetes + normal glucose) using logistic regression, as these two groups are what have been compared in other studies?

I have the following comments:

1. Avoid the use of the word `risk` for your study – you study associations with your cross-sectional study.
2. Line 38 – a better and more recent reference to diabetes prevalence is from the IDF 2021 Diabetes Atlas and the associated journal references
3. Line 30 – there are already prospective longitudinal studies … and even one on BPS.
4. Lines 55, 60 – provide references here
5. Line 62 – these meta-analyses are not recent
6. Line 103 – it would be helpful to provide a flow chart as a Supplementary Figure. How many people were actually included in the logistic regression models given that there would be missing covariate data (impute this missing data? – see below).
7. Give the name, address of the laboratory where the bisphenols were measured. I am surprised that there is no author from the laboratory.
8. Line 121 - How was urinary creatinine taken into account? this is mentioned for models, line 162 but are the concentrations of the three variables, reported in Figure 1, already adjusted for creatinine?
9. You appear to have left-censored data – given that there is a LOD for all three variables. Is regression analysis on continuous variables appropriate or would working in quartile groups be more appropriate? What are the percentages of participants at the LOD for the three variables? In the Fillol article (reference 20), multiple imputation was mentioned for left-censored values of biomarkers, with a general reference to Stata and Royston 2014. How was this carried out in practice, because the data are not missing at random.
10. Line 142 – you give `n (%)` not `proportions` in the tables
11. Line 144 – you indicate that when >5% data was missing, a missing class was added – but how did you use this for continuous variables? Why did you not use multiple imputation for missing covariates?
12. Line 150 – more importantly than dispersion, the bisphenols have a very skewed distribution, with heavy right tails. The residuals in a regression analysis are likely to be skewed.
13. Good that you have not just assumed that the relations are linear, but you should include this fact in the results, make it more prominent. Most authors assume linearity in regression models.
14. Line 155 – give the base of the logarithm, so that others can check their ORs against your ORs
15. Do you have measures of waist circumference, for adjustment?
16. Table 1 – add a line for No diabetes, no prediabetes
17. Figure 1
    a. there is no need for the total population bar – and it is not appropriate in the text to compare levels lines 178-179, total vs a sub-group.
    b. what do you mean by `observations outside the range of adjacent values are not represented`? what is `adjacent`? usually outliers are represented by dots – if they are very extreme outliers, indicate `n observations >x` …)
    c. Provide the percentages with values <LOD.
18. Table 2
    a. I was confused by the heading of the first column – use `No diabetes, No prediabetes`
    b. Was the Welch test used –age is the only variable that is continuous
19. Line 209 – As the relation is non-linear with BPA (it is linear with log_e_BPA or log_10_BPA), the OR will differ according to the level of BPA – so to say that it is `for an increase of 0.1 μg/l in log transformed …` is not correct. It will be for a one unit increase or a 0.1 unit increase in log_e_BPA. Check/change the Legend of Table 3
20. Table 3 – Are there missing data on the covariates, in which case the unadjusted and adjusted models with be on different populations?
21. It would be good to show spline curves of the relations between the three variables and the incidence of (diabetes +prediabetes)
22. Was there any effect modification by sex, BMI as found in the D.E.S.I.R. study?
23. Line 235 – these studies are not recent …
24. Line 243 – you should mention that the D.E.S.I.R study hazard ratios used two measures of these bisphenols BPA and BPS, measured at a 3 year interval, not just one measure
25. Line 249
    a. D.E.S.I.R.
    b. Your study combined people with diabetes and prediabetes, in contrast to other studies that studied just diabetes. Your study **population** included diabetes, prediabetes and people with `normal` glucose
26. Everywhere, you should write `multivariable` models as you are predicting one variable from many variables
27. Line 288 – insufficient statistical power is not an argument, as your ORs are far from being statistically significant
