# Supplementary material for: Relation of urinary bisphenol concentration and diabetes or prediabetes in French adults: A cross-sectional study
Source: PLoS One. 2023 Mar 30;18(3):e0283444. doi: 10.1371/journal.pone.0283444 (PMC10062552; doi:10.1371/journal.pone.0283444)
Supplement: Attachment — Submitted filename: Reponse to reviewers.docx [file pone.0283444.s003.docx]

**Response to reviewers, manuscript “Relation of urinary bisphenol concentration and diabetes or prediabetes in French adults: a cross-sectional study”**

**Journal requirements:**

1.  Please ensure that your manuscript meets PLOS ONE's style requirements, including those for file naming. The PLOS ONE style templates can be found at

<https://journals.plos.org/plosone/s/file?id=wjVg/PLOSOne_formatting_sample_main_body.pdf>  and

<https://journals.plos.org/plosone/s/file?id=ba62/PLOSOne_formatting_sample_title_authors_affiliations.pdf>

Response: It has been done, and the files have been renamed.

2. Please ensure that you have specified (1) whether consent was informed and (2) what type you obtained (for instance, written or verbal, and if verbal, how it was documented and witnessed). If your study included minors, state whether you obtained consent from parents or guardians. If the need for consent was waived by the ethics committee, please include this information.

Response: Previously, it was only specified in the Esteban protocol article (reference 19). We added this sentence directly in the “Esteban study” part of the Methods: “An informed consent form for participation in the study was signed in triplicate by the participants.”

3. We note that the grant information you provided in the ‘Funding Information’ and ‘Financial Disclosure’ sections do not match.

When you resubmit, please ensure that you provide the correct grant numbers for the awards you received for your study in the ‘Funding Information’ section.

Response: The authors received no specific funding for this work, and no grants have been received. However, the French national public health agency, which is the investigator of the study, was funded by the public authorities. We added this sentence in the cover letter “The French National Public Health Agency runs the French human biomonitoring programme, which is funded by the French Ministries of Health and Environment.” We respectfully hope that these clarifications have answered all your questions.

4.  In your Data Availability statement, you have not specified where the minimal data set underlying the results described in your manuscript can be found. PLOS defines a study's minimal data set as the underlying data used to reach the conclusions drawn in the manuscript and any additional data required to replicate the reported study findings in their entirety. All PLOS journals require that the minimal data set be made fully available. For more information about our data policy, please see <http://journals.plos.org/plosone/s/data-availability>.

Upon re-submitting your revised manuscript, please upload your study’s minimal underlying data set as either Supporting Information files or to a stable, public repository and include the relevant URLs, DOIs, or accession numbers within your revised cover letter. For a list of acceptable repositories, please see <http://journals.plos.org/plosone/s/data-availability#loc-recommended-repositories>. Any potentially identifying patient information must be fully anonymized.

Important: If there are ethical or legal restrictions to sharing your data publicly, please explain these restrictions in detail. Please see our guidelines for more information on what we consider unacceptable restrictions to publicly sharing data: <http://journals.plos.org/plosone/s/data-availability#loc-unacceptable-data-access-restrictions>. Note that it is not acceptable for the authors to be the sole named individuals responsible for ensuring data access.

We will update your Data Availability statement to reflect the information you provide in your cover letter.

Reponse: The data is public but cannot be shared on the internet (open data) for ethical and legal reasons, imposed by the French regulatory authorities. That is why we specified "Due to legal and ethical concerns, the data underlying the results presented in the study are available, on justified request, from Santé publique France, the French national public health agency." Santé publique France is a public government agency that can be contacted via its website <https://www.santepubliquefrance.fr/>.

**Additional Editor Comments :**

Dear Author,

It is necessary to inform whether a normality test was applied to identify the type of parametric and non-parametric test. What was the criterion for choosing the student's t test?

Response: Dear Editor, thank you for your detailed comments and vigilance. Only the Student test was used. This was rectified in the methods and in the note of Table 2 [table 1 in the revised manuscript] (as mentioned by reviewer #1, we only describe one continuous variable in Table 2[table 1 in the revised manuscript]).

The normality test was applied to identify the statistic test to use. We also looked at the graphical aspect of the data.

Additionally, although it is a population-based study, it is important to show that the sample used for the hypothesis tested has sufficient power. In this way, calculate the power of your results against the sample used and present it at the end of the discussion. You already inform that the power is insufficient in the end, but how much would it be? And how this undermines the scientific evidence produced.

Response: Thank you for this interesting comment. In light of your contribution and also to comply with Reviewer#1’s comment number 27 (i.e. “line 288: insufficient statistical power is not an argument, as your ORs are far from being statistically significant”), we propose to remove the following sentence from the discussion. Indeed, it added confusion and was not entirely correct, as stated by Reviewer#1. We have deleted the following sentence : “Finally, although the sample size (N = 852) is reasonable compared to other international studies, the absence of a significant association between urinary BPS and BPF concentrations and the diabetic or prediabetic status may be due to an insufficient statistical power.”

Is the evidence valid for decision-making by health professionals? Talk more about this aspect in the discussion.

Response: Our evidence is valid and deserves to be transmitted to decision-makers. Although, it concerns more government decisions than day-to-day clinical decision of health professionals. We said in our paper in the conclusion that: “In light of the results of our study, this regulation of BPA seems justified”. However, the most useful studies for health policy makers are those that establish causality. We are convinced of the value of the information we are providing with this cross-sectional study, but we have nevertheless tempered the interpretation of our results, since the study design only allows us to generate or provide elements of confirmation of certain hypotheses. We have specified that: “prospective longitudinal studies are necessary needed to demonstrate a causal link between exposure to bisphenols and the onset of diabetes or prediabetes”.

**Review Comments to the Author**

Reviewer #1: It is not clear whether the data is available, as required by PLOSOne `The PLOS Data policy requires authors to make all data underlying the findings described in their manuscript fully available without restriction, with rare exception`

Response: We answered the Editor directly. The data is public but can only be available on demand, due to ethics and legal reasons.

I have responded `partly` to the question `Is the manuscript technically sound, and do the data support the conclusions?` as I am not sure of some of the statistical analyses, and I expect the authors to respond to my comments in the attached review document. I indicate above that the statistics have been performed correctly.

Response: We answered every comment in the attached document (“Response to reviewers”).

Reviewer #2: Congratulations to the authors for conducting the study and reporting the results in this manuscript.
The manuscript is concise and of pleasant to read.
Some contributions:
1. Describe in the method the exclusion criteria of the participants - this information was presented only in the results.

Response: Thank you for the comment. We clarified the exclusion criteria in the study population paragraph in the method. The new paragraph is as follows:

“To study the relationship between bisphenol exposure and the risk of diabetes or prediabetes, Esteban participants had to be at least 18 years. Exclusion criteria were: absence of at least one questionnaire (interview-guided and self-administered), absence of medical examination, having gestational diabetes, absence of fasting serum glucose measurement and absence of measurement of urinary concentration of BPA, BPS, and BPF.”

2. The results presented in Table 1 do not contribute to answer the research question. I suggest removing these results and going straight to the results presented in Table 2.

Response: Table 1 describes the total populations of the Esteban study (n=2345) and the population of our study (n=852). I agree that it is not essential, however it does support that the two population present similar characteristics. We propose to remove it from the main manuscript but add it in a Supporting information section.

3. As it is a cross-sectional study, it is better to refer to the odds ratio and not the risk.

Response: Thank you for this point. We agree and thought we tried to make this clear in the first draft of the manuscript. We have re-read the manuscript with this particular point in mind. We don’t use the word risk anymore and have made changes throughout the manuscript.

4. The first paragraph of the conclusion is general and does not add information to the reader. I suggest starting with the text of the second paragraph.

Response: Thank you, we agree. We started the conclusion with the second paragraph, and enrich the last paragraph with some elements of the first paragraph that was deleted.

The last paragraph became: “In addition, as diabetes is a major public health concern and environmental pollutants may be involved in the etiology of diabetes, prospective longitudinal studies are still needed to demonstrate a causal link between exposure to bisphenols and the onset of diabetes or prediabetes.”

________________________________________________________________________________________________________________________________________________________________________________________________________________________________________________________________________________________________________________________________________

Reviewer #1:

This is a cross-sectional study of the association between the bisphenols BPA, BPS and BPF with (diabetes + prediabetes) in 852 adults living in France. The authors find a detrimental association for BPA but no association for BPS nor for BPF.

Do you get the same results if you study just diabetes, prediabetes compared to `normal glucose tolerance`, using multinomial regression; or for diabetes vs (prediabetes + normal glucose) using logistic regression, as these two groups are what have been compared in other studies?

Response: Thank you for your comments. These would have been interesting sensitivity analyses. However, given the small number of diabetics with bisphenol measurements (n=62) and the number of adjustment factors, we did not consider it relevant to perform the analysis by separating the diabetic state from the pre-diabetic state.

I have the following comments:

1. Avoid the use of the word `risk` for your study – you study associations with your cross-sectional study.

Response : Thank you for this point. We agree and thought we tried to make this clear in the first draft of the manuscript. We have re-read the manuscript with this particular point in mind. We don’t use the word risk anymore and have made changes throughout the manuscript.

2. Line 38 – a better and more recent reference to diabetes prevalence is from the IDF 2021 Diabetes Atlas and the associated journal references

Response : Thank you for the needed update, we added the following sentence “It was estimated to be 10.5% in 2021, rising to 12.2% in 2045, according to the International Diabetes Federation” and cited “Sun H, Saeedi P, Karuranga S, Pinkepank M, Ogurtsova K, Duncan BB, Stein C, Basit A, Chan JCN, Mbanya JC, Pavkov ME, Ramachandaran A, Wild SH, James S, Herman WH, Zhang P, Bommer C, Kuo S, Boyko EJ, Magliano DJ. IDF Diabetes Atlas: Global, regional and country-level diabetes prevalence estimates for 2021 and projections for 2045. Diabetes Res Clin Pract. 2022 Jan;183:109119. doi: 10.1016/j.diabres.2021.109119. Epub 2021 Dec 6. PMID: 34879977.”

3. Line 30 – there are already prospective longitudinal studies … and even one on BPS.

Response: Thank you, indeed they are already few longitudinal studied. However, more information is still needed. We have changed the sentences in the conclusions of the manuscript and the abstract to better reflect the reality of the state of research and not to imply that there are not already some longitudinal studies.

Manuscript “[…]analysis of prospective longitudinal studies are still needed to demonstrate a causal link between exposure to bisphenols and the onset of diabetes or prediabetes.”

Abstract “However, analysis of prospective longitudinal studies are still necessary to demonstrate a causal link between bisphenol exposure and the risk of diabetes or prediabetes.”

4. Lines 55, 60 – provide references here

Response: We already cited the papers, but later in the paragraph. We cited the paper at the end of the sentences indicated, and added the reference “Alonso-Magdalena, P.; Morimoto, S.; Ripoll, C.; Fuentes, E.; Nadal, A. The Estrogenic Effect of Bisphenol A Disrupts Pancreatic Beta-Cell Function in Vivo and Induces Insulin Resistance. Environmental health perspectives 2006, 114 (1), 106–112. <https://doi.org/10.1289/ehp.8451>.” to flesh out the point.

5. Line 62 – these meta-analyses are not recent

Response: Time flies, it’s true that 2015 and 2016 is not that recent. We scratched the word.

6. Line 103 – it would be helpful to provide a flow chart as a Supplementary Figure. How many people were actually included in the logistic regression models given that there would be missing covariate data (impute this missing data? – see below).

Response: We did not provide a flow chart but we provided detailed numbers corresponding to the inclusion and exclusion criteria in the first paragraph of the results. In addition, we clarified the sentence in the Study population part of the Method. The sentence became “To study the relationship between bisphenol exposure and the risk of diabetes or prediabetes, Esteban participants had to be at least 18 years. Exclusion criteria were: absence of at least one questionnaire (interview-guided and self-administered), absence of medical examination, having gestational diabetes, absence of fasting serum glucose measurement and absence of measurement of urinary concentration of BPA, BPS, and BPF.”

Concerning the missing covariate data, we responded in detail in your comments 11 and 20.

7. Give the name, address of the laboratory where the bisphenols were measured. I am surprised that there is no author from the laboratory.

Response: The analyses of bisphenols were performed by Laberca/Oniris, Nantes, France. All procedures were written according to ISO-17025 quality assurance guidelines. They were associated with the previous publication which presented all bisphenols analytical results (reference number 19 in the revised version of the manuscript: “Exposure of the French population to bisphenols, phthalates, parabens, glycol ethers, brominated flame retardants, and perfluorinated compounds in 2014–2016: Results from the Esteban study. Environment International 2021. Fillol et al.”).

8. Line 121 - How was urinary creatinine taken into account? this is mentioned for models, line 162 but are the concentrations of the three variables, reported in Figure 1, already adjusted for creatinine?

Response: Urinary creatinine concentrations were introduced into the models after logarithmic transformation (in Table 3[table 2 in the revised manuscript]). The concentrations of BPA, BPS and BPF in Figure 1 were not already adjusted for creatinine.

9. You appear to have left-censored data – given that there is a LOD for all three variables. Is regression analysis on continuous variables appropriate or would working in quartile groups be more appropriate? What are the percentages of participants at the LOD for the three variables? In the Fillol article (reference 20), multiple imputation was mentioned for left-censored values of biomarkers, with a general reference to Stata and Royston 2014. How was this carried out in practice, because the data are not missing at random.

Response:

We had given a lot of thought to how to take bisphenols into account in the model. That's why we used fractional polynomials. These did not identify a better equation to represent the relationship between bisphenols and the diabetic/pre-diabetic stage than a linear relationship.

The graphical appearance of the relationship did not point towards a quartile consideration either.

All participants have a dosage above the LOD for BPA, BPS and BPF. We had already indicated this information in the method but it was not clearly visible. We highlighted it by moving the sentence just after the definition of the LOD and LOQ values for bisphenols.

Moreover, we did not need to impute the data, as we had values for all individuals.

10. Line 142 – you give `n (%)` not `proportions` in the tables

Response: Thank you, we replaced “proportions” by “numbers and percentages” and modified the sentence as following: “Numbers and percentages were described for qualitative variables”.

11. Line 144 – you indicate that when >5% data was missing, a missing class was added – but how did you use this for continuous variables? Why did you not use multiple imputation for missing covariates?

Response: They were only two continuous variables: the age variable had no missing data, and the creatinine variable had less than 5% missing data.

Concerning multiple imputation for missing variables, this concerns only 2 variables, which are adjustment variables. We have therefore chosen to use a simpler method, taking into account the presence of missing data by adding a "missing" category.

12. Line 150 – more importantly than dispersion, the bisphenols have a very skewed distribution, with heavy right tails. The residuals in a regression analysis are likely to be skewed.

Response: Thank you for this very accurate comment. We added this information in the Methods, as follows: “To limit their dispersion as well as their skewed distribution, the bisphenol concentration values were log-transformed (with base 10)”.

13. Good that you have not just assumed that the relations are linear, but you should include this fact in the results, make it more prominent. Most authors assume linearity in regression models.

Response: Thank you. In line with your advice, in the Statistical analysis part of the method, we modified the sentence presenting this analysis. The sentence is the following “The relationships between the three bisphenols and diabetes or prediabetes were explored by fractional polynomials to determine if they should be considered continuously or not.” We do not present the result of this analysis in the methodology.

In addition, in the results, we added the following sentence to make our analysis more prominent: “The relationships between the three bisphenols and diabetes or prediabetes were explored by fractional polynomials. Based on the results of this analysis (*results not shown*), urinary bisphenol concentrations were considered continuously.”

14. Line 155 – give the base of the logarithm, so that others can check their ORs against your ORs

Response: Thank you for your vigilance, we had not thought to specify this. We used a base 10 logarithm. The precision has been added in the text as follows: “To limit their dispersion as well as their skewed distribution, the bisphenol concentration values were log-transformed (with base 10).”

15. Do you have measures of waist circumference, for adjustment?

Response: The waist circumference was measured in the study but we chose to use the body mass index.

16. Table 1 – add a line for No diabetes, no prediabetes

Response: The line was added to Table 1[Supplemental information table in the revised manuscript].

17. Figure 1
    a. there is no need for the total population bar – and it is not appropriate in the text to compare levels lines 178-179, total vs a sub-group.
    b. what do you mean by `observations outside the range of adjacent values are not represented`? what is `adjacent`? usually outliers are represented by dots – if they are very extreme outliers, indicate `n observations >x` …)
    c. Provide the percentages with values <LOD.

Response:

a. Ok, we have followed your advice and removed the three total population bars as well as the comparison in the text. The new figure will be uploaded and the results phase in the text has been modified as follows: “BPA levels are higher in participants with diabetes or prediabetes compared to people without diabetes or prediabetes, while BPS and BPF levels are similar across the two groups.”

b. We agree that the term "adjacent values" is not very clear. We have therefore removed it and left only the definition of vertical bars, which we have improved as follows: “The ends of the extended vertical lines indicate the 1st quartile minus 1.5 times the IQR and the 3rd quartile plus 1.5 times the IQR ;”.

We have chosen not to graphically represent the outliers so that the graph remains readable. We have therefore followed your advice and mentioned the number of outliers in the legend of the figure as follows: “observations outside this range are not represented (BPA ° 59 observations >6.1 °° 11 observations >7.8 / BPS ° 80 observations >1.7 °° 22 observations >1.3 BPF ° 52 observations > 0.7 °° 15 observations > 0.7; no extreme low values were observed)”.

c. All participants had a dosage above the LOD and the LOQ for BPA, BPS and BPF. We had already indicated this information in the method but it was not clearly visible. We highlighted it by moving the sentence just after the definition of the LOD and LOQ values for bisphenols.

18. Table 2
    a. I was confused by the heading of the first column – use `No diabetes, No prediabetes`

Response: Thank you, we modified this in Table 2[table 1 in the revised manuscript].

b. Was the Welch test used –age is the only variable that is continuous

Response: Thank you for your vigilance. Only the Student test was used. This was rectified in the methods and in the note of Table 2. You had spotted it well, we only describe one continuous variable in Table 2.

19. Line 209 – As the relation is non-linear with BPA (it is linear with log_e_BPA or log_10_BPA), the OR will differ according to the level of BPA – so to say that it is `for an increase of 0.1 μg/l in log transformed …` is not correct. It will be for a one unit increase or a 0.1 unit increase in log_e_BPA. Check/change the Legend of Table 3

Response: Yes, the legend was modified to correspond to the analysis. It was already correctly stated in the title of the Table 3[table 2 in the revised manuscript]. The legend is as following: “‡BPA: log-transformed bisphenol A, BPS: log-transformed bisphenol S, BPF: log-transformed bisphenol F”.

20. Table 3 – Are there missing data on the covariates, in which case the unadjusted and adjusted models with be on different populations?

Response: There are some missing data in the adjustment variables, but they are few. We have specified the number of people in each model in Table 3. n =852 for the unadjusted model and n=806 for the adjusted model.

21. It would be good to show spline curves of the relations between the three variables and the incidence of (diabetes +prediabetes)

Response: Thank you for this interesting comment and proposal. This was discussed among the co-authors, but we preferred to focus on the results of the models, and therefore did not add any further analysis or graphics.

However, we have still highlighted the analyses concerning the link between BPS, BPA and BPF and (diabetes+prediabetes), as per your advice in comment 13, by adding a sentence to the results, as mentioned in our response to the said comment.

22. Was there any effect modification by sex, BMI as found in the D.E.S.I.R. study?

Response: Stratification analysis by sex and BMI was very interesting in the D.E.S.I.R. study. Unfortunately, here we did not want to perform such analysis here because of the small number of people who had a bisphenol measurement and who were diabetic.

23. Line 235 – these studies are not recent …

Response: Ok, we scratched the word “recent”.

24. Line 243 – you should mention that the D.E.S.I.R study hazard ratios used two measures of these bisphenols BPA and BPS, measured at a 3 year interval, not just one measure

Response: This fact is indeed important. It was already mentioned in the discussion, with the sentence: “In addition, the detection (≥ LOD) of BPS glucuronide in urine at baseline, after 3 years, or at both time points was associated with incident diabetes (HR = 2.81 [95% CI: 1.74-4.53]).”

25. Line 249
    a. D.E.S.I.R.

Response: Thank you, it was modified in the manuscript.

b. Your study combined people with diabetes and prediabetes, in contrast to other studies that studied just diabetes. Your study **population** included diabetes, prediabetes and people with `normal` glucose

Response: We clarified the sentence. The sentence is now the following “The results of our BPA study are consistent with the results of the D.E.S.I.R. study, but in a population with a larger age group and more recent years, and with the inclusion of the notion of pre-diabetes in our analyses.”.

26. Everywhere, you should write `multivariable` models as you are predicting one variable from many variables

Response: The modifications were made throughout the manuscript.

27. Line 288 – insufficient statistical power is not an argument, as your ORs are far from being statistically significant

Response: Thank you for this interesting comment. Indeed, this sentence (line 288 of the previous version of the manuscript) added confusion and was not entirely correct, so we have deleted it.
