# Supplementary material for: Relation of urinary bisphenol concentration and diabetes or prediabetes in French adults: A cross-sectional study
Source: PLoS One. 2023 Mar 30;18(3):e0283444. doi: 10.1371/journal.pone.0283444 (PMC10062552; doi:10.1371/journal.pone.0283444)
Supplement: Attachment — Submitted filename: Response to Reviewers.docx [file pone.0283444.s004.docx]

**Response to reviewers, manuscript “Relation of urinary bisphenol concentration and diabetes or prediabetes in French adults: a cross-sectional study”**

**Journal requirements:**

Please review your reference list to ensure that it is complete and correct. If you have cited papers that have been retracted, please include the rationale for doing so in the manuscript text, or remove these references and replace them with relevant current references. Any changes to the reference list should be mentioned in the rebuttal letter that accompanies your revised manuscript. If you need to cite a retracted article, indicate the article’s retracted status in the References list and also include a citation and full reference for the retraction notice.

Response: It has been done.

**Review Comments to the Author**

Reviewer #1: Thank you for your responses to my comments. The paper is easy to read, well documented.

I still have some comments on the revised manuscript.

1. Line 16 – it should be `prevalence` not `risk`

Thank you for the comment. We replaced ‘risk’ by ‘prevalence’.

2. Line 20 and elsewhere in the manuscript, you should write `multivariable models` not `multivariate models`.

Response: Thank you for this point. We changed ‘multivariate models’ to ‘multivariable models’ in lines 20, 160, 261 and 266.

3. Line 22 you quote a `percentage` not a `proportion`

Response: We replaced the term ‘proportion’ with ‘percentage’.

4. line 24 and elsewhere in the text line 211, Table 2 legend) – this should be?: OR for an increase of 0.1 units in log transformed concentration of BPA (µg/L)

Response: Thank you for this precision, the wording has been changed.

5. `prevalence` not `risk`

Response: On line 28, we changed ‘risk’ to ‘prevalence’.

6. Line 79 add `BPS, and BPF and the prevalence of diabetes or prediabetes…`

Response: Thank you, we added ‘the prevalence of’ in the text.

7. Line 147 – you correctly use the `standard deviation` not the `standard error`. Change this.
8. Table 1 the footnote should read `standard deviation` not `standard error`

Response: Sorry for this mistake, we replaced ‘standard error’ by ‘standard deviation’ in the text and in the footnote of Table 1.

9. Line 266 - `a French population` - it is not the entire French population

Response: On line 227, we changed ‘of the French general population’ to ‘conducted on a sample of the French general population’. On line 288, we replaced ‘In our sample of the French adult population’ by ‘In our sample of the metropolitan French adult population’, the overseas territories not being included.
